# Assessing the rationale of prescribing carbapenems among hospitalized patients with documented penicillin allergy: implications for stewardship

**DOI:** 10.1017/ash.2024.5

**Published:** 2024-03-18

**Authors:** Anne-Valérie Burgener-Gasser, Jeanne Fasel, Delia Halbeisen, Karin Hartmann, Maja Weisser-Rohacek, Carole Kaufmann, Sarah Tschudin-Sutter

**Affiliations:** 1 Division of Infectious Diseases and Hospital Epidemiology, University Hospital Basel, Basel, Switzerland; 2 Division of clinical Pharmacy, University Hospital Basel, Basel, Switzerland; 3 Division of Allergy, Department of Dermatology, University Hospital Basel, Basel, Switzerland

## Abstract

**Background:**

A reported history of penicillin allergy frequently leads to the prescription of carbapenems as a substitute for penicillin to avoid allergic reactions. Such self-reported allergies need to be accurately characterized to identify targeted antibiotic stewardship interventions that potentially minimize unnecessary carbapenem use.

**Design:**

Retrospective cohort study.

**Method:**

The proportion of hospitalized patients with penicillin allergy history receiving carbapenem prescriptions was evaluated between January 1st, 2017 and December 31st, 2018 at the University Hospital Basel, Switzerland. The appropriateness of carbapenem prescription of each patient was evaluated using institutional guidelines based on previously published recommendations.

**Results:**

Our analysis revealed that among 212 patients with recorded penicillin allergy, of the 247 carbapenem treatment episodes, 79 (32%) were unjustified. Abdominal and lower respiratory tract infections were most frequently associated with inappropriate carbapenem use (OR 2.64, 95% CI 1.22–5.71, *P* = .014 and OR 2.26, 95% CI 1.08–4.73, *P* = .031). The recorded allergy type was not documented or unclear in 153 patients (72%) and penicillin allergy was only confirmed in 2 patients (0.9%). Inconsistencies in allergic symptom documentation and allergy types were found between the institution’s two software programs.

**Conclusion:**

While a multimodal approach to identify and accurately label penicillin allergies remains essential to reduce inappropriate carbapenem use, our findings highlight the need for comprehensive and easily accessible guidelines for carbapenem utilization and structured history-based allergy assessment as an initial screening tool, embedded in a tailored digital allergy record template.

## Background

Penicillin allergy is documented in 8–25% of patients’ medical records.^1–3^ Documentation is commonly based on self-reported allergies that prompt prescription of alternative classes of antibiotics to avoid allergic reactions.^4,5^ However alternative antibiotics may be less effective, incur higher healthcare costs, and potentially increase the risk for antimicrobial resistance.^6,7^ In addition with only 1–10% of them ultimately showing true allergic reactions.^2,8,9^

A small number of studies have highlighted increased prescription rates of carbapenems in patients with documented penicillin allergy. In the context of emerging resistance to carbapenems and increasingly limited treatment options, documentation of penicillin allergies without careful evaluation of the validity of the allergy history results in unnecessary carbapenem prescriptions.^6,10–12^ Given the limited research on the frequency and underlying rationales for carbapenem usage among patients with a history of penicillin allergy^4,5,13^ efforts aimed at identifying the nature of self-reported penicillin allergies may constitute an important antibiotic stewardship intervention, potentially resulting in a reduction of unjustified carbapenem use.

Therefore, in the present study of hospitalized patients with a penicillin allergy history treated with carbapenem we investigated the proportion of carbapenem treatment that was unjustified, the reasons for defining treatment as unjustified, and discuss the implications of our findings in relation to current published antibiotic stewardship interventions.

## Methods

This study was conducted at the University Hospital Basel, a tertiary care hospital in Basel, Switzerland with more than 35,000 hospital admissions annually. This was a retrospective study of medical records. The study population consisted of hospitalized patients with documented penicillin allergy who were treated with carbapenem between January 1st, 2017 and December 31st, 2018. The objective was to determine the appropriateness of carbapenem treatment in this population.

Demographic, clinical, and treatment data, allergy characteristics and prospective monitoring data on the occurrence of allergies during hospitalization were extracted from the electronic history record and exported for analysis to an anonymized study database on Research Electronic Data Capture (REDCap). The categorization of carbapenem treatment was defined as receiving at least one single dose of carbapenem. Allergy-specific data were collected from two software programs, the electronic history records (system A) and a dedicated electronic allergy database (system B). System A provides an interface for medical history, where allergies are usually recorded during admission, yet it allows ongoing documentation. System B, the electronic allergy database provides a template for clinicians to record information on drug allergies, free-text entries for symptoms experienced by the patient, allergy type, grade of reaction, and availability of an allergy card. All patients with a penicillin allergy history in system B were included and further evaluation was conducted to determine what was recorded in system A in the respective patients. The appropriateness of carbapenem prescription of each patient with a penicillin allergy history reported in systems A and B during the study period was retrospectively evaluated using our institutional guidelines based on published recommendations (Table 4)^14^ and was categorized as justified or unjustified. In this process, both empirical and definitive treatments were considered for justification. Notably, cases where carbapenem was initially given due to ESBL or AmpC colonization, yet not de-escalated following culture results despite the potential indication, were considered unjustified. Conversely, cases where carbapenem use was supported by antibiogram-guided de-escalation were considered justified. In case of discrepancies between the allergy types in the two systems, the information with the more precise description has been considered, respectively the data in favor of the more severe grade of allergy reaction was applied.

In subsequent analyses, potential discrepancies between the two documentation systems were assessed.

Descriptive analyses were performed by reporting counts and proportions, as well as medians and interquartile ranges. Univariable comparisons between patients with justified and unjustified carbapenem treatment were performed using the chi-square test (or Fisher’s exact test, where appropriate) for categorical variables and the Student’s *t*-test or the Mann Whitney-*U*-test (where appropriate) for continuous variables. The Shapiro-Wilk test was used to determine if continuous variables are normally distributed. Uni-and multivariable logistic regression analyses were performed to assess associations between unjustified carbapenem use and the most common indications for the use of antibiotics. The multivariable model included the most common indications identified in our study. The Hosmer-Lemeshow goodness-of-fit test was performed to assess adequate model fit. All analyses were performed using STATA version 15.0 (Stata Corp., College Station, Texas, USA). Two-sided *P*-values of less than or equal to .05 were considered significant. The study was approved by the local ethics committee (EKNZ-Number 2019-01268). The Strengthening the Reporting of Observational Studies in Epidemiology guidelines were followed.^15^

## Results

During the study period, 212 patients with a penicillin allergy history were treated with carbapenems at our institution. Among those, 110 (51.9%) were female, the median age was 70 years (interquartile range 55–79). The median length of the hospital stay was 13 d (interquartile range 8–24). During their hospital stay, 13 (6.1%) of the 212 patients died.

In the majority of patients (118; 55.7%), the time point of the first manifestation of penicillin allergy was unknown. In 37 (17.5%) patients, initial manifestation occurred more than ten years ago. Symptoms of allergic reactions to penicillin at first presentation were presented in 160 (75.52%) patients and were unknown in 52 (24.5%) patients (Table 1). The type of allergic reaction at initial presentation of penicillin allergy was unknown in 144 (67.9%) patients. Type I allergic reaction was documented in 39 (18.4%) patients (Figure S1 (online)). Among the 212 patients with a penicillin allergy history receiving carbapenem treatment, a penicillin allergy had been confirmed by an allergist or other specialist in only two cases (0.9%). Sixteen patients (7.6%) had received an allergy card after their first allergic reaction. There were inconsistencies in the documentation of allergic symptoms and allergy types between the two software programs used at the institution (Table 1, Figure S1 (online)).

**Table 1.** Symptoms of penicillin allergy as recorded in the two software programs (system A and system B)

	System A		System B	
Symptoms	*n* ^a^	%^b^	*n* ^a^	%^b^
Skin rash	100	47.2	37	17.5
Hives	14	6.6	6	2.8
Itching	9	4.3	1	0.5
Fever	2	0.9	2	0.9
Swelling	34	16.0	19	9.0
Shortness of breath	30	14.2	9	4.3
Watery eyes	1	0.5	1	0.5
Anaphylaxis	16	7.6	6	2.8
Nausea	4	1.9	4	1.9
Diarrhea	11	5.2	6	2.8
Other	15	7.1	4	1.9
Unknown	52	24.5	137	64.6

a Total number of patients 212

b Refer to the total number of patients in the denominator

Prospective monitoring during hospitalization identified that eighteen (8.5%) patients experienced an allergic reaction to one of the antibiotic classes during their hospital stay specified in Table 2. Both the administration of 3–4th generation cephalosporins (OR 2.47, 95% CI 1.29–3.66, *P* < .001) and piperacillin/tazobactam (OR 2.95 95% CI 1.74–4.17, *P* < .001) were associated with allergic reactions during hospitalization, of which only one manifested as anaphylactic after administration of piperacillin/tazobactam in a patient with previous documentation of an unclear reaction to penicillins (Table 2). A further assessment of their penicillin allergy after discharge was recommended for only 25 (11.8%) patients.

**Table 2.** Characteristics of allergic reaction during hospital stay

	n/median	%/IQR
Occurrence of any allergic reaction during hospital stay	18	8.5
* Causative antimicrobial*		
Flucloxacillin	1	0.5
Penicillin-Betalactamase inhibitor	6	3.3
Cephalosporins (1st/2nd generation)	2	1.1
Cephalosporins (3rd/4th generation)	5	2.4
Carbapenem	3	1.4
Fluorochinolone	1	0.5
*Recorded type of allergic reaction*		
Type I	1	0.5
Type IV	3	1.4
Intolerance	1	0.5
Unclear/unknown	13	6.1

During the study period, 491 antimicrobial treatment regimens were administered to 212 patients with a penicillin allergy history. 247 (50.3%) of these episodes involved administration of carbapenems. Of the 247 carbapenem treatment episodes, 79 (32%) were unjustified according to our local treatment guidelines (Tables 3 and 4)^14^. The median length of unjustified carbapenem use was 5 d (3–8), and 7 d (4–13) for justified use. The most common diagnoses for unjustified carbapenem use were: lower respiratory tract infection (22.8%), urogenital tract infection (22.8%), and abdominal infection (21.5%). The most common diagnosis for justified carbapenem use was urogenital tract infection (19.6%) (Table 3). None of the three most common indications were associated with unjustified carbapenem use in univariable analyses (Table S1 (online)). In multivariable analyses including the three most common indications for carbapenem use, among the most common indications, abdominal infection and lower respiratory tract infection were associated with inappropriate carbapenem use (OR 2.64, 95% CI 1.22–5.71, *P* = .014 and OR 2.26, 95% CI 1.08–4.73, *P* = .031) (Table S1 (online)). The Hosmer-Lemeshow goodness-of-fit test revealed an insignificant *P*-value indicating adequate model fit.

**Table 3.** Comparisons between episodes (n=247) with and without unjustified carbapenem use

	Unjustified carbapenem use (*n* = 79)		Justified carbapenem use (*n* = 168)		*P*-value^b^
	*n*/median	%	*n*/median	%	
*Indication for antimicrobial treatment* ^a^					0.001
Abdominal infection	17	21.5	22	13.1	
Arthritis	1	1.3	2	1.2	
Bacteremia	2	2.5	15	8.9	
Endocarditis	1	1.3	3	1.8	
Elevated markers of inflammation	3	3.8	2	1.2	
Fever	2	2.5	22	13.1	
Implant-associated infection	0	0.0	1	0.6	
ORL-infection	1	1.3	1	0.6	
Skin or soft tissue infection	6	7.6	4	2.4	
Catheter-associated infection	0	0.0	4	2.4	
Upper respiratory tract infection	0	0.0	1	0.6	
Lower respiratory tract infection	18	22.8	26	15.5	
Osteomyelitis	1	1.3	9	5.4	
Surgical site infection	1	1.3	15	8.9	
Surgical prophylaxis	8	10.1	3	1.8	
Urogenital tract infection	18	22.8	33	19.6	
CNS-infection	0	0.0	5	3.0	

ORL: Otorhinolaryngology

a All infections related to surgical interventions, regardless of the type of surgery were classified as surgical site infections.

b Overall *P*-value (across all indications)

**Table 4.** Criteria for carbapenem use^14^

1. Septic shock 2. Type of infection a. Empiric therapy i. Neutropenic fever ii. Central nervous system infection iii. Necrotizing fasciitis b. Definitive therapy i. Neutropenic fever ii. Central nervous system infections 3. Type of pathogen detected in confirmed infections: a. Extended spectrum betalactamase (ESBL)-producing Enterobacterales b. AmpC beta-lactamase building pathogens i*. Enterobacter* spp., *Klebsiella aerogenes, Serratia marcescens, Citrobacter freundii, Morganella morganii, Hafnia alvei* c. *Pseudomonas* or *Acinetobacter spp*. 4. Resistance profile of the pathogen 5. Anaphylactic reaction to a penicillin (Type I allergic reaction)

## Discussion

A substantial amount of unjustified carbapenem prescriptions has been reported from different studies around the world.^16–18^ Yet, the majority of these investigations have largely evaluated all carbapenem prescriptions issued by their institutions, without paying specific attention to patients with a history of penicillin allergy.

In this study, 32% of the carbapenem treatment episodes, did not meet the criteria for carbapenem use according to local guidelines.

The effectiveness of guidelines for appropriate carbapenem utilization in conjunction with audits and feedback has been previously demonstrated.^19,20^ In the study of *Shimata et al.* applying a carbapenem prescribing algorithm led to a 3-fold reduction in carbapenem use, improved antibiogram susceptibilities, and cost-effectiveness.^19^ A similar intervention in the research work of *Garcia-Rodriguez et al*. yielded favorable clinical outcomes, including a reduction in the incidence of hospital-acquired multidrug-resistant (MDR) bloodstream infections (BSI), as well as a decrease in 30-d all-cause crude mortality rates among patients with MDR BSI.^20^ While this study did not discuss mechanisms by which carbapenem reduction results in improvement of clinical outcome in patients with MDR BSI, the authors attribute the simultaneous decrease of candidemias to shorter antibiotic treatment, narrower-spectrum antibiotic, and less yeast colonization in patients.^20^

Documentation of penicillin allergy in medical records is associated with increased non-penicillin antibiotic treatments, higher antibiotic costs, and extended hospital stay, emphasizing the necessity for accurate history assessments^6,12,21^ In our study, the diagnosis of penicillin allergy was primarily based on history, with symptoms recorded as unknown in more than two-thirds of cases and the allergy type as unclear in 72.2% of cases, indicating insufficient quality of history-taking and -recording. Only in two cases was the allergy confirmed by a specialist.

A drug challenge dose following a negative penicillin skin test has a nearly 100% negative predictive value, but its specialized and time-consuming nature limits broader use.^22^ In contrast, a history-based approach is cost-effective, easy to implement, and does not require a specialized team.^22–24^

Various investigations have explored the precision of a history-based questionnaire for the assessment of antibiotic allergies.^25–27^ In preoperative contexts, a structured allergy history increased beta-lactam prophylaxis by 277 (57%) cases out of 485 patients, reducing alternative antibiotic use from 81.9% to 55.9%.^28^ Similarly, systematic preoperative checklist excluded IgE-mediated reactions in 337 (67%) of 503 self-reported antibiotic allergies and in a multicenter study, a clinical decision rule accurately identified low-risk penicillin allergies with a 96.3% negative predictive value.^27,29^ Given this evidence, through an accurate medical history, penicillin allergies in our study could have been more precisely characterized, potentially preventing a substantial portion of unjustified carbapenem prescriptions.

In many medical facilities, allergy data, typically documented in electronic health records (EHRs), faces challenges such as incomplete information, misclassification, and labels not removed after negative test results.^30–34^ Our hospital employs two software programs for patient history and medication prescribing, revealing inconsistencies. Accurate EHR allergy records are critical for safe antibiotic prescribing. Addressing this, recent studies propose interventions like algorithms to detect inconsistencies, text recognition system for identifying free-text and structured allergic reactions in the EHR, coded into defined reaction concepts, and integrating alerts to enhance beta-lactam allergy documentation postdrug challenge.^35–38^ To determine if such digital applications can benefit our hospital’s allergy documentation system, intervention studies will be necessary. A starting point could involve replacing free-text entries with a structured list of defined allergy symptoms, mandating allergy documentation, and providing relevant clinician training.

Our study has several limitations, including its single-center retrospective study design limiting its generalizability to other settings. However, this is a commonly accepted design for evaluating the appropriateness of drug prescriptions in a hospital’s setting. The study relied on electronic health record data, which may be incomplete or inaccurate depending on the quality of the history-taking and data entry. Thus, in the majority of patients suspected of having an allergic reaction during hospitalization, no symptom-specific data were recorded in either software program. Yet, the absence of a corresponding entry in the patient record implies that a severe allergic reaction was unlikely. Further, our study population included only patients who were marked as allergic to penicillin in our institution’s internal patient documentation and who received carbapenems during their hospital stay. It is unknown how many patients received an alternative antimicrobial treatment based on an alleged allergy, without any further documentation. Yet as treatment decisions are made relying on the information entered into the electronic patient records, our study design allows us to assess the potential impact on treatment decisions. Furthermore, we did not include patient-specific data in our multivariable analysis as the primary aim was to assess indications and their disciplines associated with inappropriate carbapenem use in order to potentially target department-specific interventions. Finally, the study did not assess the potential long-term consequences of unjustified carbapenem use, such as the development of antimicrobial resistance or adverse events.

## Conclusion

This study provides insights into the appropriateness of carbapenem prescribing among patients with documented penicillin allergy. Carbapenem treatment was not justified in a significant proportion of patients with penicillin allergy. The diagnosis of penicillin allergy was based primarily on history, in which most symptoms and allergy types were recorded as unknown and confirmation was commonly lacking. These findings highlight the need for (i) accessible and easily applicable guidelines for the rational use of carbapenems, (ii) the implementation of a systematic penicillin history checklist including penicillin risk stratification along with training of the respective clinicians, (iii) the redesign of the digital allergy template making it mandatory to fill in more detailed allergy characteristics when entering the patient history and (iv) systematic skin testing in patients with high-risk penicillin allergy.

## Supporting information

Burgener-Gasser et al. supplementary material Burgener-Gasser et al. supplementary material

## Supplementary material

For supplementary material accompanying this paper visit http://doi.org/10.1017/ash.2024.5. click here to view supplementary material
