# Supplementary material for: Assessing the rationale of prescribing carbapenems among hospitalized patients with documented penicillin allergy: implications for stewardship
Source: Antimicrob Steward Healthc Epidemiol. 2024 Mar 18;4(1):e37. doi: 10.1017/ash.2024.5 (PMC10945937; doi:10.1017/ash.2024.5)
Supplement: Burgener-Gasser et al. supplementary material [file S2732494X24000056sup001.docx]

**Table S1**: Associations between infection sites and the inappropriate antimicrobial choice.

|  | **Univariable (unadjusted) analyses** | | | **Multivariable (adjusted*) analyses**** | | |
| --- | --- | --- | --- | --- | --- | --- |
|  | OR | 95%CI | p-value | OR | 95%CI | p-value |
| Urogenital tract infection | 1.12 | 0.58-2.17 | 0.732 | 1.68 | 0.81-3.47 | 0.162 |
| Lower respiratory tract infection | 1.61 | 0.82-3.15 | 0.164 | 2.26 | 1.08-4.73 | 0.031 |
| Abdominal infection | 1.92 | 0.95-3.88 | 0.070 | 2.64 | 1.22-5.71 | 0.014 |

*including all three indications (urogenital tract infection, lower respiratory tract infection and abdominal infection)

** Hosmer-Lemeshow test: chi2=0.00, p=1.000

**Figure S1**: Numbers of patients (n=212) with recorded type of allergic reaction in system A and system B.

**System A System B**
